# Unveiling Sudden Transitions Between Classical and Quantum Decoherence in the Hyperfine Structure of Hydrogen Atoms

**DOI:** 10.3390/e27111161

**Published:** 2025-11-15

**Authors:** Kamal Berrada, Smail Bougouffa

**Affiliations:** Department of Physics, College of Science, Imam Mohammad Ibn Saud Islamic University (IMSIU), P.O. Box 90950, Riyadh 11623, Saudi Arabia; sbougouffa@imamu.edu.sa

**Keywords:** hydrogen atoms, Lindblad master equation, geometric measures, classical correlation, quantum correlation, total correlation, hyperfine Hamiltonian, 03.67.Mn, 03.65.Yz, 42.50.-p, 71.55.Jv

## Abstract

This paper investigates the dynamics of quantum and classical geometric correlations in the hyperfine structure of the hydrogen atom under pure dephasing noise, focusing on the interplay between entangled initial states and environmental effects. We employ the Lindblad master equation to model dephasing, deriving differential equations for the density matrix elements to capture the evolution of the system. The study explores various entangled initial states, characterized by parameters $a_{1}$, $a_{2}$, and $a_{3}$, and their impact on correlation dynamics under different dephasing rates Γ. A trace distance approach is utilized to quantify classical and quantum geometric correlations, offering comparative insights into their behavior. Numerical analysis reveals a transition point where classical and quantum correlations equalize, followed by distinct decay and stabilization phases, influenced by initial coherence along the *z*-axis. Our results reveal a universal sudden transition from classical to quantum decoherence, consistent with observations in other open quantum systems. They highlight how initial state preparation and dephasing strength critically influence the stability of quantum and classical correlations, with direct implications for quantum metrology and the development of noise-resilient quantum technologies. By focusing on the hyperfine structure of hydrogen, this study addresses a timely and relevant problem, bridging fundamental quantum theory with experimentally accessible atomic systems and emerging quantum applications.

## 1. Introduction

Within the framework of quantum mechanics, a key characteristic of quantum information science is the existence of quantum correlations between spatially separated quantum systems. The most established measure for quantifying genuine nonclassical correlations is quantum discord (QD), introduced by Ollivier and Zurek [1]. This entropy-based measure is particularly valuable for identifying quantum correlations in both entangled and mixed separable states [2,3]. Quantum discord is essential across multiple areas of quantum optics [4,5,6,7,8,9,10,11,12,13,14]. However, its general dynamical behavior presents significant challenges due to complex optimization processes, restricting exact solutions to specific cases [15,16,17,18]. To overcome this, a geometric approach has been employed, utilizing various distance-based methods that are relatively simpler to calculate [19,20,21,22,23,24]. Among these, the trace-norm (Schatten 1-norm) geometric measure stands out, offering a reliable and physically sound method for assessing both quantum and classical correlations [25,26]. Beyond simply measuring correlations, a critical challenge and essential foundation for cutting-edge quantum technologies is understanding how quantum and classical correlations respond to various decoherence mechanisms. Within this context, it is widely accepted that quantum correlations surpassing entanglement exhibit greater resistance to decoherence and evade the sudden loss phenomenon [27,28,29,30,31]. Both theoretical studies and experimental evidence have shown that, for specific initial states subjected to local Markovian dephasing environments, the trace-norm geometric quantum discord (GQD-1) displays intriguing behaviors, including freezing and dual abrupt shifts [32,33,34]. Furthermore, the dynamical decoupling (DD) technique has been utilized to extend the finite time period during which GQD-1 (or QD) maintains a constant value and to manage the occurrence of double (or single) sudden transitions [35,36,37,38]. However, the DD method faces limitations, notably an adverse interaction with non-Markovian dynamics, which disrupts coherence preservation [38]. Likewise, the effectiveness of the DD approach relies heavily on precise timing of pulses [36,38].

The hydrogen atom, with its elegantly simple structure, has historically served as a cornerstone of quantum mechanics understanding, offering profound insights into the behavior of electrons and nuclei in diverse physical, chemical and biological contexts [39,40,41,42]. Beyond its foundational role in quantum theory, the hydrogen atom emerges as a pivotal element in quantum information science, providing a natural system for exploring quantum correlations. The electron and nuclear spins in the hydrogen atom offer a physically intuitive framework and a well-defined Hilbert space for investigating bipartite quantum entanglement, whose entanglement, quantified by two-qubit concurrence and quantum coherence measures, can be directly linked to fundamental, constants such as the Planck constant, the Boltzmann constant, electron and proton masses, the fine-structure constant, the Bohr radius, and Bohr magneton. At low temperatures, the hyperfine structure (HFS) states of the hydrogen atom exhibit inherent entanglement, which diminishes rapidly as the temperature increases, eventually disappearing beyond a critical threshold of $\tau_{c}\approx 5.35\;\mu \mathrm{eV}$. This phenomenon is rooted in the thermal equilibrium behavior of the HFS states, where the entanglement is sensitive to the balance between the energy gap and thermal energy [43,44,45]. Recent studies have also revealed nuclear-polarized phases of hydrogen atoms embedded in solid H_2_ films [43,46], showing significant deviations from the Boltzmann distribution at low temperatures [43,44,45], raising intriguing questions about the role of quantum effects in such systems. The electron- and nuclear-spin degrees of freedom in the hydrogen atom provide a platform for studying entanglement and connecting to broader quantum information applications. Earlier research on electron-spin dynamics in two-electron double-quantum-dot systems [47,48] has demonstrated the potential of such systems as qubits for quantum information technologies [49,50,51]. Similarly, nuclear spins, particularly in systems like nitrogen-vacancy centers in diamonds, have been identified as valuable resources for quantum information processing [52,53,54,55]. In contrast to previous works that have explored electron–proton coordinate entanglement [56] or provided a formalism for HFS entanglement [57], this study uncovers a novel aspect: the ability of an external magnetic field to induce and sustain HFS entanglement even at temperatures well above the critical threshold $\tau_{c}$. This magnetically induced entanglement defies the thermal degradation typically observed in such systems, offering a new avenue for entanglement engineering in low-temperature environments, including gases and solids.

The theory of open quantum systems explores the dynamics of quantum systems coupled to their surrounding environments, a subject of significant interest since the foundational development of quantum mechanics [58]. Despite considerable theoretical progress, fundamental challenges remain unresolved, notably the phenomenon of decoherence, the dissipation of quantum coherence arising from system-environment interactions. This process has attracted substantial attention in the fields of quantum information and computation, where decoherence poses a formidable barrier to the realization of quantum information processors [59,60,61]. The maintenance of quantum coherence is indispensable for the operation of quantum computers, quantum cryptography, and quantum teleportation. Furthermore, decoherence serves as a critical mechanism for understanding the quantum-to-classical transition, wherein the emergence of classical properties from quantum systems is interpreted as a consequence of environmentally induced decoherence.

This study aims to elucidate the intricate dynamics of quantum and classical correlations within the hyperfine structure of the hydrogen atom under the influence of pure dephasing noise, with a focus on leveraging entangled initial states to probe these effects. By employing a trace distance approach to distinguish and quantify geometric correlations, we seek to uncover the mechanisms governing the observed transition between classical and quantum decoherence regimes, as influenced by initial coherence parameters and dephasing strength. While sudden transitions between classical and quantum decoherence have been extensively studied in model open quantum systems [25,26,62,63], their manifestation in fundamental atomic systems such as hydrogen has received comparatively little attention. Given that hydrogen is both a paradigmatic and experimentally accessible system, analyzing these transitions provides not only fundamental insights into the dynamics of quantum correlations but also potential implications for precision spectroscopy, quantum metrology, and the development of noise-resilient quantum technologies. Our work thus addresses a timely and relevant question, drawing parallels with universal behaviors observed in other open quantum systems and offering guidance for controlling correlation stability in practical atomic quantum devices.

The structure of this paper is outlined as follows: Section 2 develops the theoretical groundwork by examining the hyperfine interaction and the significance of entangled initial states. This section presents the Lindblad master equation for dephasing, derives the differential equations for the matrix elements, and details the system’s evolution modeling under dephasing noise, achieving analytic solutions for various entangled states characterized by $a_{1}\left(0\right)$, $a_{2}\left(0\right)$, and $a_{3}$. Section 3 investigates a trace distance approach for assessing and comparing classical and quantum geometric correlations. Section 4 provides a comprehensive analysis that integrates the previous sections, focusing on the shift from classical to quantum decoherence regimes and the effects of initial coherence. Finally, Section 5 encapsulates the conclusions, their relevance to quantum metrology and noise-resistant technologies, and proposes avenues for future investigation.

## 2. Hyperfine Interaction and Entangled States in Hydrogen

### 2.1. Hyperfine Structure of the Hydrogen Atom

The hyperfine interaction in the hydrogen atom originates from the coupling between the intrinsic magnetic moments of the electron and the proton. In the ground electronic state (1s), the orbital angular momentum vanishes (ℓ=0), implying that no orbital magnetic field acts on the nucleus. Consequently, the hyperfine interaction is purely magnetic dipole–dipole in nature, determined solely by the electron and proton spins [42].

This spin–spin coupling gives rise to the celebrated 21cm line (frequency 1420MHz), which plays a central role in both atomic physics and astrophysics [64]. Theoretically, the interaction can be described within first-order perturbation theory by a Hamiltonian proportional to the scalar product of the electron and proton spin operators:

$$H_{\mathrm{int}}\propto I\cdot S,$$

where I and S denote the nuclear (proton) and electronic spin operators, respectively. In the presence of an external magnetic field, this Hamiltonian must be extended to incorporate the Zeeman interaction [64,65,66].

For a spin-$\frac{1}{2}$ electron and a spin-$\frac{1}{2}$ proton, the effective hyperfine Hamiltonian is expressed in terms of Pauli operators as

(1) $$H_{\mathrm{HF}}=J\;\sigma_{e}\cdot \sigma_{p}=J\left(\sigma_{x}^{\left(e\right)}\sigma_{x}^{\left(p\right)}+\sigma_{y}^{\left(e\right)}\sigma_{y}^{\left(p\right)}+\sigma_{z}^{\left(e\right)}\sigma_{z}^{\left(p\right)}\right),$$

where *J* is the hyperfine coupling constant, while $\sigma_{e}=\left(\sigma_{x}^{\left(e\right)},\sigma_{y}^{\left(e\right)},\sigma_{z}^{\left(e\right)}\right)$ and $\sigma_{p}=\left(\sigma_{x}^{\left(p\right)},\sigma_{y}^{\left(p\right)},\sigma_{z}^{\left(p\right)}\right)$ act on the electron and proton Hilbert spaces, respectively.

An explicit expression for *J* is given by [64]:

(2) $$J=\frac{2\pi }{3}\frac{1}{4\pi \varepsilon_{0}}\cdot \frac{g_{e}e}{2m_{e}}\cdot \frac{g_{p}e}{2m_{p}}\cdot \frac{\hbar^{2}}{c\pi a_{0}^{3}},$$

where $\varepsilon_{0}$ is the vacuum permittivity, $a_{0}$ the Bohr radius, *ℏ* the reduced Planck constant, and *c* the speed of light. The quantities $g_{e}$, $g_{p}$, $m_{e}$, and $m_{p}$ represent the *g*-factors and masses of the electron and proton, respectively.

Each spin-$\frac{1}{2}$ degree of freedom resides in a two-dimensional Hilbert space spanned by

$$H_{e}={\{|\;↑}_{e}{\rangle ,|\;↓}_{e}\rangle \},\;H_{p}={\{|\;↑}_{p}{\rangle ,|\;↓}_{p}\rangle \}.$$

The combined spin space is thus

$$H=H_{e}⊗H_{p}={\{|\;↑}_{e}{↑}_{p}{\rangle ,|\;↑}_{e}{↓}_{p}{\rangle ,|\;↓}_{e}{↑}_{p}{\rangle ,|\;↓}_{e}{↓}_{p}\rangle \}.$$

Diagonalization of $H_{\mathrm{HF}}$ yields a singlet state

(3) $$|S\rangle =\frac{1}{\sqrt{2}}\left({|\;↑}_{e}{↓}_{p}{\rangle -|\;↓}_{e}{↑}_{p}\rangle \right),\;E_{S}=-3J,$$

and a triplet manifold

(4) $$\begin{array}{cccc} |T_{+}\rangle & ={|\;↑}_{e}{↑}_{p}\rangle ,\; & E_{T_{+}} & =J, \end{array}$$

(5) $$\begin{array}{cccc} |T_{0}\rangle & =\frac{1}{\sqrt{2}}\left({|\;↑}_{e}{↓}_{p}{\rangle +|\;↓}_{e}{↑}_{p}\rangle \right),\; & E_{T_{0}} & =J, \end{array}$$

(6) $$\begin{array}{cccc} |T_{-}\rangle & ={|\;↓}_{e}{↓}_{p}\rangle ,\; & E_{T_{-}} & =J. \end{array}$$

The hyperfine splitting between the singlet and triplet manifolds is

$$\Delta E=E_{T}-E_{S}=4J\approx 5.88\;\mu \mathrm{eV},$$

where $E_{T}$ denotes the common triplet energy. In the absence of an external magnetic field, the ground state is the maximally entangled singlet |S〉. The application of a magnetic field lifts the degeneracy among the triplet states, enabling external control of the hyperfine energy structure, which is critical for quantum information applications.

### 2.2. Quantum Dynamics Under Pure Dephasing

In open quantum systems, *decoherence* broadly refers to the loss of quantum coherence due to interactions with the environment, which can involve both phase and energy relaxation. A specific form of decoherence, *pure dephasing*, occurs when no energy exchange takes place and only the relative phases between quantum states are randomized.

Dephasing is a central decoherence mechanism in quantum systems, where environmental noise disrupts phase coherence between quantum states while leaving populations (energy-level occupations) unchanged. In the context of the hydrogen atom’s hyperfine interaction, this form of noise arises naturally from coupling to external fields or fluctuations that act differently on the spin components of the electron and proton. To model such decoherence accurately, we employ the Lindblad formalism with local $\sigma_{z}$-type Lindblad operators, which selectively suppress coherences without inducing energy relaxation. This approach provides a clear and tractable framework for analyzing how entanglement and quantum coherence evolve under phase noise in the two-qubit (electron–proton) system.

Importantly, pure dephasing allows us to isolate the phase-noise-induced dynamics of correlations, which is essential for observing and characterizing the sudden transitions between classical and quantum decoherence regimes. By controlling the initial state and the dephasing strength, our model captures the interplay between classical and quantum correlations in a transparent and experimentally relevant setting, providing insights into both fundamental decoherence processes and potential applications in quantum metrology and noise-resilient quantum technologies.

The evolution of the density matrix ρ(t) is governed by the Lindblad equation:

(7) $$\frac{d\rho }{dt}=-i\left[H_{\mathrm{HF}},\rho \right]+D\left(\rho \right),$$

where $H_{\mathrm{HF}}$ is the hyperfine Hamiltonian and D(ρ) is the dissipator that encodes the environmental interaction. For pure dephasing, we consider the following local Lindblad operators:

(8) $$L_{e}=\sigma_{z}^{e}⊗I_{p},\;L_{p}=I_{e}⊗\sigma_{z}^{p},$$

with corresponding dephasing rates $\gamma_{e}$ and $\gamma_{p}$. The dissipator becomes:

(9) $$D\left(\rho \right)=\gamma_{e}\left(L_{e}\rho L_{e}-\rho \right)+\gamma_{p}\left(L_{p}\rho L_{p}-\rho \right),$$

which simplifies due to the property $L_{e}^{2}=L_{p}^{2}=I$, valid for Pauli operators.

Let A=J/ℏ denote the strength of the coherent interaction. In the standard computational basis $H={\{|↑}_{e}{↑}_{p}{\rangle ,|↑}_{e}{↓}_{p}{\rangle ,|↓}_{e}{↑}_{p}{\rangle ,|↓}_{e}{↓}_{p}\rangle \}$, the evolution equations for the density matrix elements $\rho_{ij}\left(t\right)$ under dephasing are:

Populations:

(10) $$\frac{d\rho_{11}}{dt}=0,$$

(11) $$\frac{d\rho_{22}}{dt}=-2iA\left(\rho_{23}-\rho_{32}\right),$$

(12) $$\frac{d\rho_{33}}{dt}=2iA\left(\rho_{23}-\rho_{32}\right),$$

(13) $$\frac{d\rho_{44}}{dt}=0.$$

Coherences:

(14) $$\frac{d\rho_{12}}{dt}=-2iA\left(\rho_{12}-\rho_{13}\right)-2\gamma_{p}\rho_{12},$$

(15) $$\frac{d\rho_{13}}{dt}=-2iA\left(\rho_{13}-\rho_{12}\right)-2\gamma_{e}\rho_{13},$$

(16) $$\frac{d\rho_{14}}{dt}=-2\left(\gamma_{e}+\gamma_{p}\right)\rho_{14},$$

(17) $$\frac{d\rho_{23}}{dt}=-2iA\left(\rho_{22}-\rho_{33}+\rho_{23}\right)-2\left(\gamma_{e}+\gamma_{p}\right)\rho_{23},$$

(18) $$\frac{d\rho_{24}}{dt}=-2iA\left(\rho_{24}-\rho_{34}\right)-2\gamma_{e}\rho_{24},$$

(19) $$\frac{d\rho_{34}}{dt}=-2iA\left(\rho_{24}-\rho_{34}\right)-2\gamma_{p}\rho_{34},$$

with $\rho_{ij}^{*}=\rho_{ji}$. These equations explicitly capture the essential features of pure dephasing: the invariance of populations and the exponential decay of off-diagonal coherences. Importantly, the coupling between $\rho_{22}$, $\rho_{33}$, and $\rho_{23}$ shows how coherence loss directly influences population transfer between entangled spin configurations.

### 2.3. Entangled Initial States and Dynamics

To explore the evolution of quantum correlations in the hydrogen hyperfine system under Markovian open-system dynamics, we consider a class of two-qubit entangled states known as *X*-states. These states are not only physically relevant but also experimentally preparable, making them ideal candidates for quantum information processing tasks such as teleportation, quantum cryptography, and entanglement-based communication [67,68,69].

The *X*-state has the following general form, parameterized by real coefficients $a_{1},a_{2},a_{3}\in \left[-1,1\right]$ and featuring maximally mixed marginals ($\rho_{A(B)}=I/2$):

(20) $$\rho \left(0\right)=\frac{1}{4}\left(\begin{array}{cccc} 1+a_{3} & 0 & 0 & a_{1}-a_{2}\\ 0 & 1-a_{3} & a_{1}+a_{2} & 0\\ 0 & a_{1}+a_{2} & 1-a_{3} & 0\\ a_{1}-a_{2} & 0 & 0 & 1+a_{3} \end{array}\right).$$

This form encompasses both maximally entangled Bell states ($|a_{1}\left|=\right|a_{2}\left|=\right|a_{3}|=1$) and Werner states ($|a_{1}\left|=\right|a_{2}\left|=\right|a_{3}|=a$) as special cases, allowing for a comprehensive investigation of correlation dynamics.

Within the Markovian framework, modeled using the Lindblad master equation, the time-evolved state ρ(t) retains the X-form:

(21) $$\rho \left(t\right)=\left(\begin{array}{cccc} \rho_{11}\left(t\right) & 0 & 0 & \rho_{14}\left(t\right)\\ 0 & \rho_{22}\left(t\right) & \rho_{23}\left(t\right) & 0\\ 0 & \rho_{32}\left(t\right) & \rho_{33}\left(t\right) & 0\\ \rho_{41}\left(t\right) & 0 & 0 & \rho_{44}\left(t\right) \end{array}\right),$$

as supported by Equations (10) and (14). This structure is particularly advantageous for analytical treatments of entanglement dynamics and quantum discord, especially in the presence of spin-selective decoherence channels characteristic of atomic systems.

For clarity and completeness, the full analytical expressions for the time-dependent density matrix elements, corresponding to several representative and physically relevant initial states, are presented here. Nonzero elements:

(22) $$\rho_{11}\left(t\right)=\rho_{44}\left(t\right)=\frac{1}{4}\left(1+a_{3}\right),$$

(23) $$\rho_{22}\left(t\right)=\rho_{33}\left(t\right)=\frac{1}{4}\left(1-a_{3}\right),$$

(24) $$\rho_{14}\left(t\right)=\frac{1}{4}\left(a_{1}-a_{2}\right)e^{-2\Gamma t},$$

(25) $$\rho_{23}\left(t\right)=\frac{1}{4}\left(a_{1}+a_{2}\right)e^{-2\Gamma t}.$$

Zero elements:

(26) $$\begin{array}{cc} \rho_{12}\left(t\right) & =\rho_{13}\left(t\right)=\rho_{24}\left(t\right)=\rho_{31}\left(t\right)=\rho_{34}\left(t\right)=0, \end{array}$$

with $\rho_{ij}=\rho_{ji}^{*}$, where $\Gamma =\gamma_{e}+\gamma_{p}$.

Having established the dynamical behavior under dephasing, we now turn to the study of trace distance classical and quantum correlations, followed by a comparative analysis.

## 3. Trace Distance Approach to Classical and Quantum Correlations: Comparative Insights

In this section, we provide a detailed account of the trace-norm geometric quantifiers that are widely employed to investigate quantum and classical correlations in bipartite systems. These measures are particularly insightful in the context of two-qubit systems, where analytical results are available and can be directly linked to the underlying dynamics of decoherence and information flow.

In the current model, the electron and proton spins create a bipartite system through the coupling induced by the hyperfine interaction. This spin-spin coupling acts as the foundation of the correlations linking the two subsystems. At first, the hyperfine interaction generates quantum correlations, or entanglement, between the spins. When exposed to pure dephasing noise, the off-diagonal elements of the joint density matrix diminish, thereby decreasing quantum correlations while maintaining statistical correlations among the spin populations. These statistical correlations represent classical correlations, highlighting the shared population structure dictated by the hyperfine coupling even after entanglement ceases.

Let ρ denote the density matrix of a bipartite system *A*–*B*. The quantification of correlations is based on comparing ρ with two special reference states. The first is the closest classical–quantum state, denoted $\rho_{c}$, which retains only classical correlations and is obtained by performing a complete local projective measurement on subsystem *A*. The second is the product state of local marginals, $\pi =\rho_{A}⊗\rho_{B}$, which represents a completely uncorrelated reference.

The 1-norm (trace-norm) geometric measures of quantum and classical correlations, $Q_{G}\left(\rho \right)$ and $C_{G}\left(\rho \right)$, are defined as [25,26]

(27) $$Q_{G}\left(\rho \right)=\min_{\left\{\Pi_{i}^{A}\right\}}∥\rho -\rho_{c}{∥}_{1},\;C_{G}\left(\rho \right)=\max_{\left\{\Pi_{i}^{A}\right\}}{∥\rho_{c}-\pi ∥}_{1},$$

where the trace norm is given by

(28) $${∥M∥}_{1}=\mathrm{Tr}\;\left[\sqrt{M^{†}M}\right].$$

The minimization (maximization) runs over all local projective measurements $\left\{\Pi_{i}^{A}\right\}$ on subsystem *A*. The corresponding post-measurement (classical–quantum) state is

(29) $$\rho_{c}=\sum_{k}\nu_{k}\;\Pi_{A}^{k}⊗\rho_{B}^{k},$$

with probabilities $0\leq \nu_{k}\leq 1$ and $\sum_{k}\nu_{k}=1$. The operators $\left\{\Pi_{A}^{k}\right\}$ denote a complete set of orthogonal projectors on subsystem *A*, while $\rho_{B}^{k}$ are the corresponding conditional state of subsystem *B*. From an operational perspective, $Q_{G}\left(\rho \right)$ quantifies the minimal “distance” between ρ and its closest classical approximation $\rho_{c}$, thereby capturing the intrinsically quantum part of the correlations (closely related to quantum discord). Conversely, $C_{G}\left(\rho \right)$ measures the largest distinguishability between the classical state $\rho_{c}$ and the uncorrelated reference $\pi =\rho_{A}⊗\rho_{B}$, and thus characterizes the strength of purely classical correlations.

For two-qubit systems, particularly those described by *X*-shaped density matrices such as the Bell-diagonal states introduced in Equation (20), these measures acquire closed-form expressions [32]:

(30) $$Q_{G}\left(\rho \right)=\mathrm{mid}\{|a_{1}\left|,\right|a_{2}\left|,\right|a_{3}|\},\;C_{G}\left(\rho \right)=\max\{|a_{1}\left|,\right|a_{2}\left|,\right|a_{3}\left|\right\},$$

where mid selects the intermediate value among the absolute correlation coefficients $\left\{|a_{1}|,|a_{2}|,|a_{3}|\right\}$. This result highlights the complementarity of quantum and classical parts: while the strongest correlation (the maximum) is attributed to classical correlations, the intermediate value captures the genuinely quantum contribution. The smallest correlation coefficient, although not appearing directly in these measures, still influences entanglement properties.

The dephasing process preserves the Bell-diagonal structure of such states, allowing the temporal behavior of the correlation measures to remain analytically tractable. Explicitly, one obtains

(31) $$Q_{G}\left[\rho \left(t\right)\right]=\mathrm{mid}\{|a_{1}\left(t\right)|,|a_{2}\left(t\right)|,|a_{3}\left(t\right)|\},\;C_{G}\left[\rho \left(t\right)\right]=\max\{|a_{1}\left(t\right)|,|a_{2}\left(t\right)|,|a_{3}\left(t\right)\left|\right\},$$

with time-dependent correlation coefficients evolving as

(32) $$a_{1}\left(t\right)=a_{1}e^{-2\Gamma t},\;a_{2}\left(t\right)=a_{2}e^{-2\Gamma t},\;a_{3}\left(t\right)=a_{3}.$$

The exponential suppression governed by the dephasing rate Γ reflects the irreversible loss of phase coherence in the system. Since all coefficients decay at the same rate, the relative ordering among $\left\{|a_{1}|,|a_{2}|,|a_{3}|\right\}$ can change during the dynamics. Such changes directly influence the behavior of $Q_{G}\left[\rho \left(t\right)\right]$ and $C_{G}\left[\rho \left(t\right)\right]$, which may exhibit sudden transitions in their temporal evolution, a phenomenon often referred to as the sudden-change effect.

It is also instructive to introduce the sum of the geometric measures,

(33) $$T_{G}\left[\rho \left(t\right)\right]=Q_{G}\left[\rho \left(t\right)\right]+C_{G}\left[\rho \left(t\right)\right],$$

which provides a geometric estimate of the total correlations.

This framework therefore not only provides explicit and computable formulas for quantum and classical correlations but also reveals subtle features of correlation dynamics that go beyond entanglement. Notably, even when entanglement has vanished due to decoherence, the geometric quantum discord $Q_{G}\left(\rho \right)$ can persist, highlighting the robustness of quantum correlations. At the same time, the interplay between $Q_{G}\left(\rho \right)$ and $C_{G}\left(\rho \right)$ illustrates how correlations are redistributed between the quantum and classical sectors under dephasing noise. By introducing the total geometric correlation, $T_{G}\left(\rho \right)=Q_{G}\left(\rho \right)+C_{G}\left(\rho \right)$, one obtains a direct measure of the overall correlation content in the system. Collectively, these trace distance quantifiers provide a powerful framework to probe the structure and temporal evolution of correlations in open quantum systems, forming the basis for the comparative analysis developed in the following section.

Having established the trace distance framework for quantifying classical and quantum correlations, we now turn to a numerical investigation of their dynamical behavior within the hyperfine structure model of the hydrogen atom under pure dephasing, in order to connect the theoretical formalism with physically relevant scenarios.

## 4. Numerical Analysis and Discussions

**Figure 1:**
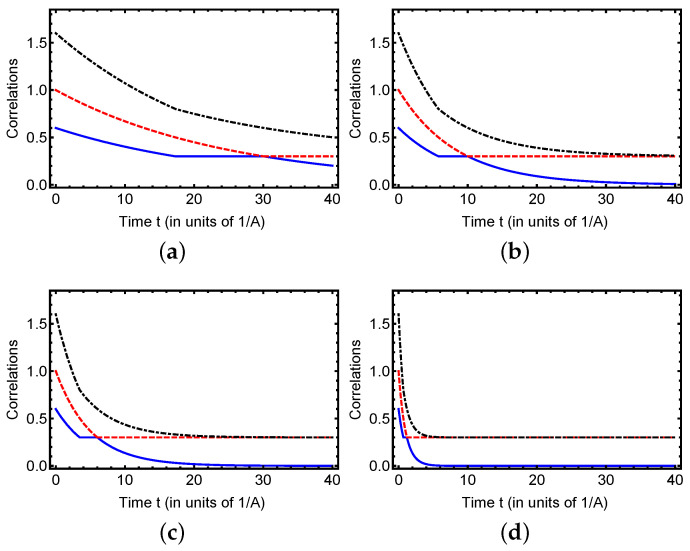
Dynamics of classical correlation (red dashed line), quantum discord (blue solid line), and the total geometric correlation (black dash-dotted line) as a function of time *t* for initial conditions $a_{1}\left(0\right)=1$, $a_{2}\left(0\right)=-0.6$, and $a_{3}=0.3$. The subfigures (**a**–**d**) illustrate the time evolution of these correlations under varying dephasing parameter Γ, corresponding to Γ=0.02A, Γ=0.06A, Γ=0.1A, and Γ=0.5A, respectively, highlighting the impact of increasing decoherence on quantum and classical correlations.

The dynamics of classical geometric correlation $C_{G}$ (red dashed line), quantum geometric correlation $Q_{G}$ (blue solid line), and total geometric correlation $T_{G}$ (black dash-dotted line) in the hyperfine structure model of the hydrogen atom under dephasing noise, as depicted in Figure 1, provide valuable insights into the evolution of correlation measures. The analysis is based on two sets of initial conditions: (a, b) with $a_{1}\left(0\right)=1$, $a_{2}\left(0\right)=-0.6$, and $a_{3}=0.6$, and (c, d) with $a_{1}\left(0\right)=1$, $a_{2}\left(0\right)=-0.3$, and $a_{3}=0.3$, under the dephasing rates Γ=0.02A and Γ=0.1A, where *A* is the hyperfine splitting constant. Under weak dephasing, Γ=0.02A (Figure 1a), the quantum correlation $Q_{G}$ initially decreases, but then exhibits a plateau over an extended time interval. This plateau indicates a regime where quantum correlations are relatively robust against decoherence, reflecting the partial preservation of off-diagonal coherences in the hyperfine system. Meanwhile, classical correlations $C_{G}$ start to decay more steadily til the transition point where both measures become equal; then the quantum geometric correlation decreses exponentially and the classical geometric stabilizations over the future time. The total correlation $T_{G}$ consistently remains above both components, providing an upper bound for the sum of quantum and classical correlations. Increasing the dephasing parameter to Γ=0.06A (Figure 1b) accelerates the decay of both correlations. Notably, the plateau in $Q_{G}$ becomes shorter and less pronounced, and the crossover point where $Q_{G}$ falls below $C_{G}$ occurs earlier, signaling a faster redistribution of correlations from quantum to classical sectors. This highlights how decoherence not only reduces the magnitude of quantum correlations but also shifts the temporal window in which they dominate over classical correlations. At Γ=0.1A (Figure 1c), the decay becomes nearly exponential for both $C_{G}$ and $Q_{G}$, with $T_{G}$ forming the upper envelope, reflecting a Markovian-like loss of coherence where populations are preserved but superpositions rapidly decay. Under strong dephasing, Γ=0.5A (Figure 1d), the correlations collapse almost immediately, demonstrating the quantum-to-classical transition, where the system effectively projects onto decoherence-preferred pointer states. Interestingly, $T_{G}$ retains a slight residual tail, confirming its role as a robust witness of total correlation even when individual components vanish.

These results illustrate the differing sensitivities of quantum and classical geometric correlations in the hydrogen hyperfine system: geometric quantum correlation is more fragile but can survive weak dephasing, while classical geometric correlations decay more uniformly. The inclusion of the total geometric correlation $T_{G}\left(t\right)$ provides a clear picture of the overall content of the correlation and its redistribution between the quantum and classical parts. This analysis lays the groundwork for exploring more complex scenarios, such as anisotropic dephasing or multi-qubit hyperfine networks, to further investigate the interplay of correlations in atomic quantum systems.

**Figure 2:**
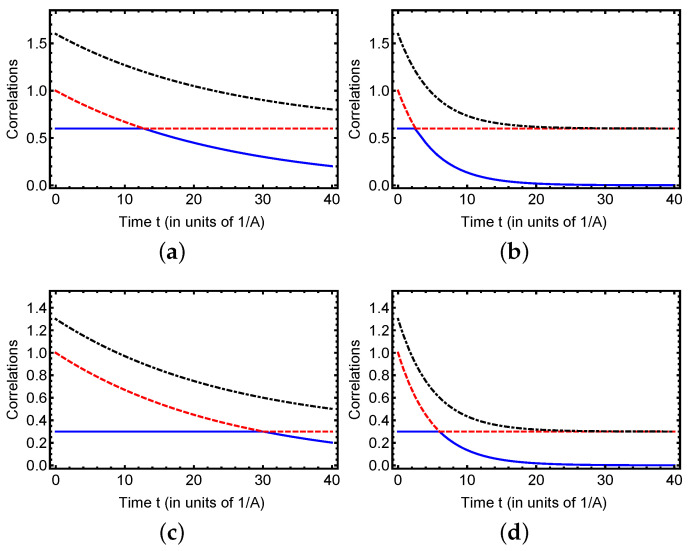
Dynamics of classical correlation (red dashed line), quantum discord (blue solid line), and quantum mutual information (black dash-dotted line) as a function of time *t* for different initial conditions. Subfigures (**a**,**b**) depict the time evolution of these correlations under dephasing rates Γ=0.02A and Γ=0.1A, respectively, with initial conditions $a_{1}\left(0\right)=1$, $a_{2}\left(0\right)=-0.6$, and $a_{3}=0.6$. Subfigures (**c**,**d**) show the same correlations under dephasing rates Γ=0.02A and Γ=0.1A, respectively, with initial conditions $a_{1}\left(0\right)=1$, $a_{2}\left(0\right)=-0.3$, and $a_{3}=0.3$.

The dynamics of classical geometric correlation $C_{G}$ (red dashed line), quantum geometric correlation $Q_{G}$ (blue solid line), and total geometric correlation $T_{G}$ (black dash-dotted line) in the hyperfine structure model of the hydrogen atom under dephasing noise, as depicted in Figure 2, provide valuable insights into the evolution of correlation measures. The analysis is based on two sets of initial conditions: (a, b) with $a_{1}\left(0\right)=1$, $a_{2}\left(0\right)=-0.6$, and $a_{3}=0.6$, and (c, d) with $a_{1}\left(0\right)=1$, $a_{2}\left(0\right)=-0.3$, and $a_{3}=0.3$, under dephasing rates Γ=0.02A and Γ=0.1A, where *A* is the hyperfine splitting constant. In Figure 2a, $Q_{G}$ (quantum geometric correlation) initially exhibits a plateau at a saturation point of approximately 0.6, extending from t=0 to t≈12 units of 1/A, after which it decreases exponentially. $C_{G}$ (classical geometric correlation) starts a decay from an initial value larger than that of $Q_{G}$, and both correlations meet at a transition point (around t≈12 in units of 1/A), where they become equal. Beyond this point, $C_{G}$ stabilizes at a reduced level over time, while $Q_{G}$ begins its exponential decay. $T_{G}$ (total geometric correlation) tracks this overall decline, reflecting the combined evolution of its quantum and classical components under weak dephasing. In subfigure (c) (some initial conditions as (a), but Γ=0.1A), $Q_{G}$ displays a plateau at an initial saturation point of approximately 0.3, lasting from t=0 to t≈30 units of 1/A, showing a longer stability duration compared to (a), then followed by an exponential decay. $C_{G}$ starts an exponential decay from a higher initial value than $Q_{G}$, and they intersect at a transition point (around t≈30 in units of 1/A), after which $C_{G}$ stabilizes at a lower value over time, while $Q_{G}$ undergoes exponential decay. $T_{G}\left(t\right)$ follows a similar downward trajectory, mirroring the behavior of both $Q_{G}\left(t\right)$ and $C_{G}\left(t\right)$ as dephasing gradually erodes the total correlation content. The higher initial $a_{3}=0.6$ in (a) sustains a shorter yet more robust plateau in $Q_{G}$ (quantum geometric correlation), reaching up to 0.6, whereas the lower $a_{3}=0.3$ in (c) enables a longer but less pronounced plateau, stabilizing around 0.3. This behavior aligns with the pivotal role of initial coherence along the *z*-axis, where a greater $a_{3}$ enhances the plateau’s strength but shortens its duration, while a reduced $a_{3}$ extends its temporal range at a lower amplitude. Meanwhile, $C_{G}$ (classical geometric correlation) undergoes a monotonic decay from its initial peak to the transition point, after which it stabilizes at a reduced level, and $T_{G}$ (total geometric correlation) follows a similar decline, reflecting the dependence of the total correlation on both components. In subfigure (b), with Γ=0.1A, $Q_{G}$ (quantum geometric correlation) exhibits a plateau similar to that in (a) (Γ=0.02A), stabilizing at approximately 0.6, but this plateau is shortened, extending from around t=0 to t≈3 units of 1/A (compared to 0 to 12 in (a)). This indicates that the increased dephasing compresses the stable regime while preserving the initial value dictated by $a_{3}=0.6$. $C_{G}$ (classical geometric correlation) decays monotonically from its initial peak to transition point, after which it stabilizes at a reduced level, and $T_{G}\left(t\right)$ (total geometric correlation) follows a similar decline, reflecting the total correlation’s dependence on both components under enhanced noise. In panel (d), with Γ=0.1A and $a_{3}=0.3$, $Q_{G}$ exhibits behavior similar to that in (c) (Γ=0.02A), reaching a steady state around 0.3. However, the plateau period is reduced, spanning approximately from t=0 to t≈6 units of 1/A, as opposed to the 0 to 30 range in (c). Despite the lower initial value of $a_{3}$, the plateau persists longer than in (b), aligning with the pattern seen at Γ=0.02A. Nevertheless, the increased dephasing shortens its duration. Meanwhile, $C_{G}$ shows a consistent decline up to the transition point, then stabilizes at a lower level, and $T_{G}\left(t\right)$ follows this trend, underscoring the noise’s influence on correlation dynamics.

The observed plateau in $Q_{G}$ under weaker dephasing (Γ=0.02A) suggests a potential control mechanism tied to the preparation of the initial state. The magnitude of $a_{3}$, which reflects the initial alignment of the spin state along the quantization axis, appears to govern the height and duration of the plateau. A higher $a_{3}$ (as in (a)) enhances the robustness of quantum correlations against dephasing, possibly by aligning the system closer to a decoherence-free subspace within the hyperfine levels. Controlling this plateau could involve optimizing the initial $a_{3}$ value to maximize coherence preservation, potentially through precise state preparation techniques such as optical pumping or microwave driving to adjust the electron–proton spin configuration. Additionally, modulating the dephasing rate Γ via environmental shielding (e.g., reducing magnetic field inhomogeneities) could extend the plateau’s temporal extent, offering a pathway to exploit quantum correlations in quantum information processing, and quantum metrology or atomic clock applications based on the hydrogen 21 cm transition.

In summary, the figure illustrates the differential sensitivity of quantum and classical correlations to dephasing. The plateau in $Q_{G}$ highlights a regime of partial coherence preservation, and its controllability provides a practical handle to enhance the lifetime of quantum correlations in atomic systems under environmental noise.

On the other hand, the transition between classical geometric correlation $C_{G}\left(t\right)$ and quantum geometric correlation $Q_{G}\left(t\right)$ in the hyperfine structure of the hydrogen atom, as observed in the provided figures, can be understood as a manifestation of the interplay between intrinsic spin dynamics and environmental dephasing. In the hyperfine model, the interaction between the electron and proton spins, governed by the Hamiltonian $H=AS_{e}\cdot S_{p}$, establishes initial correlations parameterized by $a_{1}\left(0\right)$, $a_{2}\left(0\right)$, and $a_{3}$. The dephasing noise, with rates Γ, introduces phase randomization that preferentially affects the off-diagonal coherences, which are critical to quantum correlations. Initially, $C_{G}$ dominates due to the robust classical alignment of spins, but as dephasing erodes these coherences, $Q_{G}$ becomes prominent until the transition point where $C_{G}(\bar{t}=Q_{G}\left(\bar{t}\right)$, which gives $\bar{t}=\frac{1}{2\Gamma }\ln\left(|\frac{a_{i}\left(0\right)}{a_{3}}|\right),\;i=1,2$. Beyond this point, the stabilization of $C_{G}$ reflects the preservation of classical spin orientations, while $Q_{G}$ decays exponentially as quantum superpositions are lost, driven by the environmental interaction. This transition bears similarity to phenomena observed in other open quantum systems, as detailed in Refs. [14,62,63,70,71,72]. In that study, the dynamics of quantum discord and classical correlations under nondissipative decoherence reveal an analogous behavior: for certain initial states, quantum correlations remain stable for $t<\bar{t}$, while classical correlations decay during this period. Beyond the transition time $\bar{t}$, classical correlations stabilize, and only quantum correlations are subsequently lost due to environmental interactions. This sudden shift from a classical to a quantum decoherence regime at $\bar{t}$ mirrors the crossover in the hydrogen hyperfine system, where the transition point marks a switch from classical correlation decay to quantum correlation dominance, followed by their respective stabilization and decay phases.

The origin of this transition in the hyperfine context likely stems from the competition between the coherent spin-spin coupling and the dephasing-induced noise. The initial state’s $a_{3}$ parameter, reflecting *z*-axis coherence, influences the duration and height of the $Q_{G}$ plateau, suggesting that the transition timing ($\bar{t}$) is modulated by the initial preparation and the dephasing strength Γ. This universality across systems, including the qubit pairs in [62,71] and the hydrogen atom, indicates a general feature of open quantum systems under nondissipative decoherence, where the interplay of initial conditions and environmental coupling dictates the classical-quantum correlation dynamics.

It is important to note that decoherence and dephasing are basis-dependent phenomena. In the present study, the $\sigma_{z}$-type Lindblad operators define the spin quantization axis as the relevant basis, and the observed sudden transition between classical and quantum correlations arises specifically from pure dephasing dynamics along this axis. This behavior is distinct from that reported in our previous work [73], where we analyzed general dissipative effects on quantum coherence and purity in hydrogen atoms without isolating phase-only contributions. While the detailed quantitative features of the transition depend on the initial state, the qualitative phenomenon of a sudden change between classical and quantum correlations persists across different entangled and partially entangled initial states within the dephasing basis [74].

## 5. Conclusions

In conclusion, this investigation has elucidated the evolution of classical and quantum correlations in the hyperfine structure of a hydrogen atom subjected to pure dephasing. By modeling the system’s dynamics via the Lindblad master equation, the analytic solutions of the differential equations are obtained for a particular initial X-state. Using the trace distance approach to classical and quantum correlations, we identified a critical transition point where classical and quantum correlations equalize, followed by their characteristic decay and stabilization. A key finding is the deterministic role of initial coherence along the quantization axis (parameterized by $a_{3}$) on the quantum correlation plateau: higher $a_{3}$ values produce more robust but shorter-lived plateaus, whereas lower values result in longer-lived plateaus of diminished magnitude under various dephasing rates. This transition, indicative of a sudden shift between decoherence regimes, aligns with universal behaviors in open quantum systems. Our results demonstrate that correlation stability can be controlled through initial state preparation and dephasing rate modulation, providing a foundational strategy for enhancing quantum metrological protocols and engineering noise-resilient quantum information technologies.

## Data Availability

The original contributions presented in this study are included in the article. Further inquiries can be directed to the corresponding author.

## References

[1] Ollivier H., Zurek W.H. (2001). Quantum Discord: A Measure of the Quantumness of Correlations. Phys. Rev. Lett..

[2] Rulli C.C., Sarandy M.S. (2011). Global Quantum Discord in Multipartite Systems. Phys. Rev. A.

[3] Lanyon B.P., Jurcevic P., Hempel C., Gessner M., Vedral V., Blatt R., Roos C.F. (2013). Experimental Quantum Computing without Entanglement. Phys. Rev. Lett..

[4] Dakić N., Lipp Y.O., Ma X., Ringbauer M., Kropatschek S., Barz S., Paterek T., Vedral V., Zeilinger A., Brukner C. (2012). Quantum Discord as Resource for Quantum Computation. Nat. Phys..

[5] Modi K., Cable H., Williamson M., Vedral V. (2011). Quantum Correlations in Mixed-State Metrology. Phys. Rev. X.

[6] Girolami D., Tufarelli T., Adesso G. (2013). Quantum Discord for General Two-Qubit States: Analytical Progress. Phys. Rev. Lett..

[7] Girolami D., Souza A.M., Giovannetti V., Tufarelli T., Filgueiras J.G., Sarthour R.S., Soares-Pinto D.O., Oliveira I.S., Adesso G. (2014). Quantum Discord Determines the Interferometric Power of Quantum States. Phys. Rev. Lett..

[8] Werlang T., Trippe C., Ribeiro G.A.P., Rigolin G. (2010). Quantum Correlations in Spin Chains at Finite Temperatures and Quantum Phase Transitions. Phys. Rev. Lett..

[9] Streltsov A., Kampermann H., Bruss D. (2011). Quantum Cost for State Discrimination and Classical Correlations. Phys. Rev. Lett..

[10] Adesso G., D’Ambrosio V., Nagali E., Piani M., Sciarrino F. (2014). Characterizing Quantum Correlations via Local Quantum Uncertainty. Phys. Rev. Lett..

[11] Bradler K., Wilde M.M., Vinjanampathy S., Uskov D.B. (2010). Lower Bounds on Quantum Discord. Phys. Rev. A.

[12] Gu M., Chrzanowski H.M., Assad S.M., Symul T., Modi K., Ralph T.C., Vedral V., Lam P.K. (2012). Experimental Quantum Discord in Gaussian States. Nat. Phys..

[13] Streltsov A., Zurek W.H. (2013). Quantum Discord and Work Extraction. Phys. Rev. Lett..

[14] Bera A., Das T., Sadhukhan D., Roy S.S., Sen(De) A., Sen U. (2017). Quantum Discord and Its Allies: A Review of Recent Progress. Rep. Prog. Phys..

[15] Ali M., Rau A.R.P., Alber G. (2010). Quantum Discord for Two-Qubit X States. Phys. Rev. A.

[16] Giorda P., Paris M.G.A. (2010). Gaussian Quantum Discord. Phys. Rev. Lett..

[17] Adesso G., Datta A. (2010). Quantum versus Classical Correlations in Gaussian States. Phys. Rev. Lett..

[18] Li B., Wang Z.-X., Fei S.-M. (2011). Quantum discord and geometry for a class of two-qubit states. Phys. Rev. A.

[19] Modi K., Paterek T., Son W., Vedral V., Williamson M. (2010). Unified View of Quantum and Classical Correlations. Phys. Rev. Lett..

[20] Dakić B., Vedral V., Brukner Č. (2010). Necessary and Sufficient Condition for Nonzero Quantum Discord. Phys. Rev. Lett..

[21] Bellomo B., Giorgi G.L., Galve F., Lo Franco R., Compagno G., Zambrini R. (2012). Nonlocal Memory Effects and Quantum Correlations. Phys. Rev. A.

[22] Silva I.A., Girolami D., Auccaise R., Sarthour R.S., Oliveira I.S., Bonagamba T.J., de Azevedo E.R., Soares-Pinto D.O., Adesso G. (2013). Quantum Discord in NMR Systems. Phys. Rev. Lett..

[23] Spehner D., Orszag M. (2013). Geometric Quantum Discord with Bures Distance. New J. Phys..

[24] Bromley T.R., Cianciaruso M., Lo Franco R., Adesso G. (2014). Solving Quantum Discord for Two-Qubit X States. J. Phys. A Math. Theor..

[25] Nakano T., Piani M., Adesso G. (2013). Tight Bounds for Geometric Discord. Phys. Rev. A.

[26] Paula F.M., Hor-Meyll M.O., de Oliveira T.R., Sarandy M.S. (2013). Classical-Quantum States and Geometric Correlations. Phys. Rev. A.

[27] Werlang T., Souza S., Fanchini F.F., da Silva M.G.A.P. (2009). Sudden Transition in Quantum Discord Dynamics. Phys. Rev. A.

[28] Wang B., Xu Z.-Y., Chen Z.-Q., Feng M. (2010). Quantum Correlation Dynamics in Two-Qubit Systems. Phys. Rev. A.

[29] Ferraro A., Aolita L., Cavalcanti D., Cucchietti F.M., Acín A. (2010). Almost All Quantum States Have Nonclassical Correlations. Phys. Rev. A.

[30] Yu T., Eberly J.H. (2009). Sudden Death of Entanglement. Science.

[31] Campbell S. (2013). Quantum Correlations in Spin Chains. Quantum Inf. Process..

[32] Aaronson B., Lo Franco R., Adesso G. (2013). Comparative Investigation of the Freezing Phenomena for Quantum Correlations under Nondissipative Decoherence. Phys. Rev. A.

[33] Paula F.M., da Silva M.G.A.P., Farias O.J., Souza A.M., de Azevedo E.R., Sarthour R.S., Saguia A., Oliveira I.S., Soares-Pinto D.O., Adesso G. (2013). Nearest-Neighbor Classical Correlations Reveal Quantum Discord in Critical Spin Systems. Phys. Rev. Lett..

[34] Montealegre J.D., Paula F.M., Saguia A., Sarandy M.S. (2013). Quantum Correlations and Coherence in Spin Chains at Finite Temperatures. Phys. Rev. A.

[35] Li J.-Q., Cui X.-L., Liang J.-Q. (2015). Dynamical Behavior of Quantum Discord in Two-Qubit Systems with Dephasing. Ann. Phys..

[36] Wu W., Luo D.-W., Xu J.-B. (2014). Dynamics of Quantum Correlations under Decoherence. J. Appl. Phys..

[37] Zhang J.-S., Chen A.-X. (2014). Quantum Correlation Dynamics in Open Spin Systems. J. Phys. B.

[38] Addis C., Karpat G., Maniscalco S. (2015). Quantum Discord and Non-Markovian Dynamics. Phys. Rev. A.

[39] Bohr N. (1913). On the Constitution of Atoms and Molecules. Philos. Mag..

[40] Bethe H., Salpeter E. (1957). Quantum Mechanics of One- and Two-Electron Atoms.

[41] Series G.W. (1957). Spectrum of Atomic Hydrogen.

[42] Landau L.D., Lifshitz E.M. (1977). Quantum Mechanics: The Basic Concepts.

[43] Sheludiakov S., Ahokas J., Haar F., Pentikäinen M., Bougouffa S. (2019). High-Precision Measurements of Spin Relaxation in Solid Hydrogen. Phys. Rev. Lett..

[44] Ahokas J., McDonald J.J., Jones R.O., Conradi M.S. (2006). Electron Spin Dynamics in Solid Hydrogen at Low Temperatures. Phys. Rev. Lett..

[45] Ahokas J., McDonald J.J., Jones R.O., Conradi M.S. (2010). Electron Spin Relaxation in Solid Hydrogen. Phys. Rev. B.

[46] Bigelow N.P., Freed J.H., Lee D.M. (1989). Spin-Lattice Relaxation of Electrons in Solid Hydrogen. Phys. Rev. Lett..

[47] Johnson A.C., Petta J.R., Marcus C.M., Hanson M.P., Gossard A.C. (2005). Triplet-Singlet Spin Relaxation via Nuclei in a Double Quantum Dot. Nature.

[48] Petta J.R., Johnson A.C., Taylor J.M., Laird E.A., Yacoby A., Lukin M.D., Marcus C.M., Hanson M.P., Gossard A.C. (2005). Coherent Manipulation of Coupled Electron Spins in Semiconductor Quantum Dots. Science.

[49] Taylor J.M., Engel H.A., Dür W., Yacoby A., Marcus C.M., Zoller P., Lukin M.D. (2005). Fault-Tolerant Architecture for Quantum Computation Using Electrically Controlled Semiconductor Spins. Nat. Phys..

[50] Ladd T.D., Jelezko F., Laflamme R., Nakamura Y., Monroe C., O’Brien J.L. (2010). Quantum Computers. Nature.

[51] Bennett C.H., DiVincenzo D.P. (2000). Quantum Information and Computation. Nature.

[52] Childress L., Gurudev Dutt M.V., Taylor J.M., Zibrov A.S., Jelezko F., Wrachtrup J., Hemmer P.R., Lukin M.D. (2006). Coherent Dynamics of Coupled Electron and Nuclear Spin Qubits in Diamond. Science.

[53] Gurudev Dutt M.V., Childress L., Jiang L., Togan E., Maze J., Jelezko F., Zibrov A.S., Hemmer P.R., Lukin M.D. (2007). Quantum Register Based on Individual Electronic and Nuclear Spin Qubits in Diamond. Science.

[54] Neumann P., Mizuochi N., Rempp F., Hemmer P., Watanabe H., Yamasaki S., Jacques V., Gaebel T., Jelezko F., Wrachtrup J. (2008). Multipartite Entanglement Among Single Spins in Diamond. Science.

[55] Fuchs G.D., Dobrovitski V.V., Toyli D.M., Jelezko F., Awschalom D.D. (2009). Gigahertz Dynamics of a Strongly Driven Single Quantum Spin. Science.

[56] Tommassini P., Timmermans E., de Toledo Piza A.F.R. (1998). Relaxation and Decoherence in Two-Level Quantum Systems. Am. J. Phys..

[57] Zhu G., Du K., Li Y. (2005). Decoherence and Classical Correlation in a Two-Level System Coupled to an Environment. Phys. A.

[58] von Neumann J. (1955). Mathematical Foundations of Quantum Mechanics.

[59] Nielsen M.A., Chuang I.L. (2000). Quantum Computation and Quantum Information.

[60] Benatti F., Floreanini R. (2003). Open Quantum Systems.

[61] Preskill J. (1998). Quantum Information and Computation.

[62] Mazzola L., Piilo J., Maniscalco S. (2010). Sudden Transition Between Classical and Quantum Decoherence. Phys. Rev. Lett..

[63] Xu J.S., Xu X.Y., Li C.F., Zhang C.J., Zou X.B., Guo G.C. (2010). Experimental Investigation of Classical and Quantum Correlations Under Decoherence. Nat. Commun..

[64] Demtröder W. (2010). Atoms, Molecules and Photons.

[65] Pethick C.J., Smith H. (2008). Bose–Einstein Condensation in Dilute Gases.

[66] Maleki Y., Sheludiakov S., Khmelenko V.V., Scully M.O., Lee D.M., Zheltikov A.M. (2021). Natural and Magnetically Induced Entanglement of Hyperfine-Structure States in Atomic Hydrogen. Phys. Rev. A.

[67] Bennett C.H., Wiesner S.J. (1992). Communication via One- and Two-Particle Operators on Einstein-Podolsky-Rosen States. Phys. Rev. Lett..

[68] Bouwmeester D., Pan J.-W., Mattle K., Eibl M., Weinfurter H., Zeilinger A. (1997). Experimental Quantum Teleportation. Nature.

[69] Furusawa A., Sørensen J.L., Braunstein S.L., Fuchs C.A., Kimble H.J., Polzik E.S. (1998). Unconditional Quantum Teleportation. Science.

[70] He Q.L., Xu J.B., Yao D.X., Zhang Y.Q. (2011). Sudden Transition Between Classical and Quantum Decoherence in Dissipative Cavity QED and Stationary Quantum Discord. Phys. Rev. A.

[71] García-Pérez G., Rossi M.A., Maniscalco S. (2020). IBM Q Experience as a Versatile Experimental Testbed for Simulating Open Quantum Systems. npj Quantum Inf..

[72] Hou X.W. (2024). Frozen Discord for Three Qubits in a Non-Markovian Dephasing Channel. Phys. A.

[73] Berrada K., Bougouffa S. (2025). Quantum Coherence and Purity in Dissipative Hydrogen Atoms: Insights from the Lindblad Master Equation. Entropy.

[74] Berrada K., Bougouffa S. (2025). Harnessing Quantum Entanglement and Fidelity in Hydrogen Atoms: Unveiling Dynamics Under Dephasing Noise. Appl. Sci..

